# Middle-ear effusion in children with cleft palate: congenital or acquired?

**DOI:** 10.1017/S0022215122000093

**Published:** 2022-02

**Authors:** F Kraus, R Hagen, W Shehata-Dieler

**Affiliations:** Department of Oto-Rhino-Laryngology, Plastic, Aesthetic and Reconstructive Head and Neck Surgery, University of Würzburg, Würzburg, Germany

**Keywords:** Cleft Palate, Hearing Loss, Eustachian Tube, Otitis Media With Effusion

## Abstract

**Objective:**

Cleft palates are one of the most common congenital malformations. Because of the loss of Eustachian tube function, middle-ear ventilation is reduced. The aim of this study was to determine if middle-ear effusions were present at birth or at the three-month audiological evaluation.

**Method:**

A total of 53 children with a cleft palate were included. Data review included the results of newborn hearing screening, microscopic findings, a tympanometry, a free field audiometry and intra-operative findings.

**Results:**

A total of 58.4 per cent of patients had a median, 26.4 per cent had a bilateral, 11.3 per cent had a unilateral and 3.8 per cent had a limited soft palate cleft. Newborn hearing screening showed a pass in 83.1 per cent of newborns bilaterally. The first ear microscopy showed a bilateral middle-ear effusion in 90.6 per cent of cases. During cleft surgery, bilateral paracentesis was performed in all cases, and in 90.6 per cent middle-ear effusion was sucked out.

**Conclusion:**

The majority of children with a cleft palate do not present with middle-ear effusion at birth. It develops within several days or weeks of life.

## Introduction

Cleft palate is one of the most common congenital craniofacial malformations with an incidence of 1 in 700 newborns.^1–3^ In these children, middle-ear effusion is a well-known reason for conductive hearing loss^4^ because of the reduced middle-ear ventilation. This is treated by insertion of ventilation tubes to improve the sound conduction to the inner ear.^5^ The use and the management of ventilation tubes is an issue of debate among specialists.^6–9^

In the literature, few data are available on the period of time when middle-ear effusion in children with cleft palate occurs for the first time. An incidence of 71.9 per cent up to 97 per cent within the first months is reported.^1,4,10–17^ According to Szabo *et al*., 82 per cent of the newborns with a cleft palate pass the newborn hearing screening test. The onset of middle-ear effusion is not specified.^18^ Viswanathan *et al*. describe a hearing loss in ‘early infancy’.^19^ Flynn *et al*. showed a significantly higher prevalence of middle-ear effusion in patients with cleft palate at 1, 1.5, 3 and 5 years of age^1^ compared with patients without cleft palate. Chen *et al*. stated that middle-ear effusion in a cohort of 319 children can be measured with tympanometry at the age of 9 months.^10^ They also reported on ethnic variations showing populations of Asian and native North American descent have the highest incidence, Caucasian populations have intermediate incidence and African populations have the lowest incidence of cleft palate.^10^ However, in all these publications the earliest data on the middle ear are based on investigation one month after birth in the cleft palate clinic.

The function of the Eustachian tube depends on the tensor veli palatini muscle. Its activity is almost isometric. One main action of the tensor veli palatini muscle is to actively open the Eustachian tube.^20–24^ The physiological function depends on three different pivotal points, called hypomochlia: the pterygoid hamulus, Ostmann's fatty tissue^25^ and the medial pterygoid muscle.^26^ A sufficient tubal function depends on the complete integrity of these three hypomochlia.^27^

In Germany, newborn hearing screening has been mandatory since 2009. The automated auditory brainstem response screening with the Beraphon® (Maico, Berlin, Germany) at 35 dBnHL shows a high specificity and sensitivity.^28^ This device is used for automated auditory brainstem response - screening throughout Germany. With such a soft level of the auditory stimulus, a regular, positive automated auditory brainstem response screening (‘pass’) implies a normal tube function. This is also the case in measurements using transient evoked otoacoustic emissions (TEOAE). Hence, if middle-ear function is not normal, screening automated auditory brainstem response will be abnormal and TEOAE will be absent.

The aim of this study was to determine if middle-ear effusion was present at birth or at the three month audiological evaluation in the joint cleft clinic. This retrospective study included data of newborn hearing screening, the audiological evaluation results and the clinical findings.

## Materials and methods

The study protocol was approved by the local ethics committee (number: 20190927 01). This retrospective study included 53 children who had an appointment at the joint cleft clinic between July 2015 and December 2015 and were still under revision in 2020. According to the algorithm of the joint cleft clinic, all of them had a newborn hearing screening, an audiological evaluation within the first three months of life, a lip repair at the age of six months or a palatoplasty at one year of age. During surgery of the lip or in absence of a cleft lip at the age of six months, a microscopic examination of both ears was performed including a paracentesis and, if necessary, the insertion of ventilation tubes.

Data evaluation of the charts included the results of newborn hearing screening, the clinical findings at the first appointment within the first three months, the results of tympanometry and free field audiometry as well as the intra-operative microscopic findings. The newborn hearing screening was performed by using the Beraphon MB 11 hearing screening device (Maico, Berlin, Germany; stimulus: CE-Chirp at 35 dBnHL) or by measurement of the transient evoked otoacoustic emissions (TEOAE) (Titan®, Interacoustics, Middelfart, Denmark) within the first 14 days of life. In Germany, the TEOAE were performed with different devices. Generally, automatic brainstem evoked response audiometry was carried out with Beraphon device. The statistic was calculated using the chi-square test.

## Results

In the group of 53 children, there were 58.4 per cent (31) with a median, 26.4 per cent (14) with a bilateral, 11.3 per cent (6) with a unilateral and 3.8 per cent (2) with a soft cleft palate. A total of 41.6 per cent (22) were female and 58.4 per cent (31) were male. The palatal malformation was part of a Pierre Robin sequence in 11 children. The newborn hearing screening was performed in three children by means of transient evoked otoacoustic emissions and in 50 cases by Beraphon. A total of 83.1 per cent (44) of the 53 children passed the hearing screening bilaterally.

Bilateral middle-ear effusion was detected in 90.6 per cent (48) of the children during the first clinical evaluation session at the age of 3 months. The tympanometry showed a bilateral type B curve in 86.8 per cent (42) and a bilateral type C configuration in 13.2 per cent (6). The free field audiometry showed a median reaction at 48 dB HL (20–75 dB). In 73.6 per cent (39) of the children with a ‘pass’ in the newborn hearing screening, a middle-ear effusion was seen. Only 9.5 per cent (6) with a regular newborn hearing screening showed a ventilated middle ear. In 16.9 per cent (9), a newborn hearing screening ‘fail’ with middle-ear effusion was observed. There was no significance between the regular newborn hearing screening and the middle-ear effusion at the first investigation (*p* = 0.28, chi quadrat).

During surgery, a paracentesis was performed bilaterally in all patients. In 90.6 per cent (48) of patients, a middle-ear effusion was sucked out. The middle-ear effusion was classified as ‘serous’ in 27.1 per cent (13), ‘seromucoid’ in 45.8 per cent (22) and ‘mucoid’ in 27.1 per cent (13) of cases. In all 48 children with a middle-ear effusion, a ventilation tube was inserted. Children with an aerated middle ear did not receive a tube.

The post-operative follow up with a first appointment six weeks after tube insertion showed normal free-field reactions between 20 and 30 dB in all children. The tympanometry confirmed a patent ventilation tube with high volumes. In the study group, no sensorineural hearing loss was found.

In 2020, all of the children had a five-year follow up. All patients had intact tympanic membranes with tympanosclerosis bilaterally. All pure tone audiograms showed hearing levels between − 5 and 20 dB HL bilaterally. In 92.5 per cent (49), the tympanometry showed a type A tympanogram and in 7.5 per cent (4), it showed a type B tympanogram.

## Discussion

Sufficient pantonal hearing is required for regular speech development. The most frequent reason for peripheral hearing loss in children is middle-ear effusion causing a conductive hearing loss.^29,30^ The presence of middle-ear effusion especially influences perception of high frequencies. Therefore, the observation of children with risk factors for a middle-ear effusion is necessary.^31^

The compliance of the tympanic membrane ossicle complex is based on the middle-ear ventilation.^27^ The pressure equalisation is mainly based on the tube function realised by action of the tensor tympani and tensor veli palatini muscle.^19^ A disturbance in the tube ventilation leads to a negative pressure in the middle-ear cavity followed by a serous effusion.^30,31^ If aeration of middle-ear spaces is reduced for a longer period of time, changes in the middle-ear mucosa induce a mucoid character of these effusions. Intra-operative findings of mucoid effusions are a clear sign of a chronic ventilation problem.

Newborns with a cleft palate have a predisposition to develop middle-ear effusions because of missing tubal function. In the literature, there is a long-standing debate about the use of ventilation tubes in these patients.^6,31,32^ Ventilation tubes improve the tympanic membrane middle-ear ossicle complex compliance by drainage of the middle-ear effusion.^34^ However, ventilation tubes are known to induce tympanosclerosis and can also lead to a chronic otitis epitympanalis or mesotympanalis.^31,34,35^ Nevertheless, in order to enable a normal speech development, a chronic middle-ear effusion as well as a tympanosclerosis or chronic epitympanic or mesotympanic otitis should be treated. In this study, all patients showed hearing levels between − 5 and 20 dB HL bilaterally in the pure tone audiograms at the 5-year follow up. Indeed, all of the 106 tympanic membranes had signs of tympanosclerosis. Looking at the tympanometry approximately five years after ventilation tube insertion, the majority of the study group presented a good compliance of the tympanic membrane ossicle complex.

Very little is known about the point in time when middle-ear effusions occur in patients with cleft palate.^1^ Most of the publications report on first investigations at 3 to 12 months after birth.^4,11^ Most newborns get their newborn hearing screening during the first days at the maternity ward. The hypothesis that we sought to verify in this study was that the occurrence of middle-ear effusion in patients with cleft palate is not inborn but develops during the early days or weeks of life. A newborn hearing screening result pass (using automated auditory brainstem response or TEAOE) requires sufficient middle-ear function. Otherwise, the stimulus will be attenuated by poor middle-ear function or, for the same reason, the otoacoustic emissions generated by the outer hair cells cannot be reflected to the outer ear canal properly. Thus, a regular newborn hearing screening is sign of a regular compliance of the tympanic membrane middle-ear ossicle complex.

The data from this study show that 83.1 per cent of the newborns with cleft palate pass the bilateral newborn hearing screening successfully. These data are within the same range that Szabo *et al*. reported (82 per cent).^18^ Therefore, it can be assumed that tubal function and middle-ear ventilation are intact at the time of newborn hearing screening. On examination at the first clinical appointment within the first three months of life, the microscopy and the tympanometry show an abnormal tube ventilation with middle-ear effusion in 90.6 per cent and 86.6 per cent of cases, respectively. The free field threshold at a median of 48 dB HL gives further evidence of an existing hearing loss. During intra-operative microscopy, data on the earliest performed procedure at 6 months of life (lip repair) and latest performed at 12 months of life (palatoplasty) showed that 90.6 per cent of the children had a middle-ear effusion. In 27.1 per cent of the cases, middle-ear effusion was already ‘mucoid’, which indicates the presence of a long-standing ventilation problem. This might be a clinical sign that tube function changes over the time. However, in this cohort no tympanometry was performed with the newborn hearing screening to confirm a middle-ear effusion objectively, which is a limitation of the study. The regular newborn hearing screening during the first 14 days of life is therefore an indirect sign of a ventilated middle ear.

- Poor tube ventilation with middle-ear effusion is a typical symptom of cleft palate patients
- Data in the literature are based on investigation at one month after birth in the cleft clinic
- If middle-ear function is not normal, screening automated auditory brainstem response will be abnormal and transient evoked otoacoustic emissions will be absent
- In 44 of 53 children (83.1 per cent), a regular post-natal bilateral newborn hearing was measured
- Middle-ear effusion was detected in 90.6 per cent (48) of children at the age of 3 months
- Middle-ear effusion with tube ventilation disorder due to the cleft palate is not congenital as it develops later in life

The results of this study may be explained based on the following theory. Intrauterine, the middle-ear spaces are filled with amniotic fluid. The first scream and swallowing of the newborn produce high pressures in the upper aerodigestive tract. Referring to the mechanism of Politzer's manoeuvre, this pressure may achieve a middle-ear ventilation. In the following weeks and months, the middle ears fill up with fluid and effusion is detected at the first clinical appointment at the joint cleft clinic within the first three months while the newborn hearing screening was performed earlier under quite normal middle-ear conditions. The middle-ear effusion with tube ventilation disorder because of the cleft palate is not congenital as it develops later in life.

## Conclusion

Sufficient middle-ear ventilation is based on normal activity of the tensor tympani and tensor veli palatini muscle. Accordingly, the development of poor tube ventilation with middle-ear effusion is a typical symptom of cleft palate patients. So far, little is known about the point in time of development of poor middle-ear ventilation in these patients. The data of this study show there is a regular newborn hearing screening in 83.1 per cent which is an indirect sign of tubal patency shortly after birth. Within 3 months, at the first appointment in the joint cleft clinic, 90.6 per cent of the children show a middle-ear effusion confirmed by microscopy and tympanometry. The following poor tubal function leads to a middle-ear effusion with the effect of conductive hearing loss. In conclusion, middle-ear effusion with tubal ventilation dysfunction because of cleft palate seems to develop within the first weeks of life.
